# Management Strategies for Dry Eye Syndrome in Patients with Obesity—A Literature Review

**DOI:** 10.3390/life15071102

**Published:** 2025-07-14

**Authors:** Cosmin Victor Ganea, Călina Anda Sandu, Corina Georgiana Bogdănici, Camelia Margareta Bogdănici

**Affiliations:** 1 Doctoral School, University of Medicine and Pharmacy “Grigore T. Popa”, University Street No. 16, 700115 Iasi, Romania; cosmin-victor.ganea@umfiasi.ro; 2 Department of Ophthalmology, Faculty of Medicine, University of Medicine and Pharmacy “Grigore T. Popa”, University Street No. 16, 700115 Iasi, Romania; camelia.bogdanici@umfiasi.ro

**Keywords:** dry eye disease, tear film inflammation, ocular surface, obesity, high body index, biomarkers, diet, nutrition, physical activity

## Abstract

Tear film alterations are commonly associated with ocular pathology. The tear film plays a vital role in maintaining the optical properties of the cornea and contains essential elements required for healing and preserving the integrity of the ocular surface. As a biological fluid, the tear film is easily collected using non-invasive techniques, making it a promising candidate for analysis and often referred to as an ideal biofluid. Several studies have attempted to identify biomarkers in the tear film that could be linked to systemic or ocular disorders, with the goal of developing tools for diagnosis or even early prevention. The quality and quantity of the tear film are influenced by hormonal status, emotional experiences related to social and familial events, and the work environment. Systemic disorders are often reflected at the ocular level through alterations in the tear film. Obesity is a well-recognized public health concern, extensively studied and investigated, much like other common systemic conditions. The presence of low-grade, chronic inflammation associated with excess body weight has been validated in several studies. The strategies for preventing obesity induced dry eye disease are based on regular physical activity, maintaining adequate hydration through sufficient fluid intake, weight loss, and the supplementation of essential fatty acids. This narrative literature review aims to highlight the tear film alterations associated with obesity. The article is intended for ophthalmologists, general practitioners, nutritionists, and researchers.

## 1. Introduction

### 1.1. The Evolution of Dry Eye Syndrome Diagnosis

Dry eye syndrome was first defined in 1995 by the National Eye Institute/Industry Workshop (NEI/Industry Workshop). This working group classified dry eye syndrome from a pathophysiological perspective into two main categories: aqueous tear-deficient and evaporative states [1]. In the NEI/Industry Workshop report, the term “disorder” was used, rather than “disease”—a term later adopted in the first TFOS DEWS (Tear Film & Ocular Surface Society Dry Eye Workshop) report in 2007 to describe this condition [2].

The key contribution of the TFOS DEWS I report was the recognition of dry eye syndrome (DES) as a multifactorial disorder and its impact on redefining the initial etiological algorithm. Thus, the 2007 report replaced the term “tear deficiency,” originally used in the 1995 NEI/Industry Workshop, with “aqueous deficiency,” and reclassified evaporative causes into intrinsic and extrinsic pathways [3].

In 2015, the TFOS DEWS II report introduced the concepts of inflammation and hyperosmolarity as the key pathophysiological mechanisms that both initiate and support DES. Diagnostic algorithm updates aimed to avoid a rigid, schematic distinction between aqueous-deficient and evaporative causes. This shift was based on the understanding that any form of DES may initially result from one dominant mechanism, but ultimately, both inflammation and hyperosmolarity contribute to a self-perpetuating vicious cycle that defines the disease [4].

Furthermore, the current diagnostic algorithm acknowledges the existence of neuropathic ocular pain, which may not be accompanied by observable changes on the ocular surface. Hyperosmolarity and inflammation stimulate corneal nociceptors, transmitting pain signals centrally even in the absence of clinical signs of corneal distress. In such cases, pain management therapy may be considered [3].

Evaporative DES is the most prevalent type in the general population. The evaporative component is considered a key feature of the disease, being present in all types of DES. Without this component, tear film hyperosmolarity—a hallmark of the condition—does not occur. As a result, the latest TFOS DEWS II reports propose the use of the term “hyper-evaporative state” as a more accurate descriptor, suggesting it replace the current classification of evaporative dry eye to better reflect the underlying pathophysiological mechanism [4].

In 2017, the Asia Dry Eye Society (ADES) published a report that classified DES into three major categories: aqueous-deficient dry eye, evaporative dry eye, and dry eye due to decreased wettability. ADES supports the concept that these three categories correlate with the three layers of the tear film and are directly linked to the underlying local ocular pathology [5].

ADES advocates for a treatment approach tailored to the specific deficiency within each tear film layer. Diagnosis of DES linked to a particular layer is based on the pattern of tear film breakup observed during fluorescein staining. A line break pattern is typically seen in aqueous-deficient dry eye, while random breakup patterns are characteristics of evaporative dry eye. Unlike the TFOS DEWS II report, ADES also recognizes a type of dry eye caused by impaired surface wettability. In this type, tear film breakup appears as dimple breaks or spot breaks, which are associated with epithelial cell damage and mucin layer alterations [5].

Table 1 provides a comparative overview of the diagnostic innovations introduced by each major report on DES.

### 1.2. Definition and Diagnosis of Obesity

In 2021, the number of individuals who were overweight or obese reached 1.1 billion among adult women and 1.0 billion among adult men. A continuous upward trend was observed between 1990 and 2021, with obesity increasing by 155.1% in males and 104.9% in females. If this trajectory persists, it is projected that by 2050, there will be 3.8 billion people who are overweight or obese. The most significant increases have been recorded in China, India, and the United States. To date, no nation has succeeded in curbing this alarming phenomenon by reducing the growth rates of overweight and obesity among adults [6].

Obesity is often overlooked by many clinicians as a predisposing factor in the development of DES. While classical factors such as hormonal changes, aging, and computer vision syndrome are well recognized, a major contributor is frequently neglected: excess body weight. A high amount of adipose tissue results in an increased load of pro-inflammatory macrophages, leading to a state of systemic inflammation, which in turn negatively impacts the tear film surface. Recent studies have shown that a body fat percentage of 30% in women and over 20% in men predisposes individuals to symptoms of DES [7].

Obesity is one of the most widespread disorders of the 21st century. In addition to the Body Mass Index (BMI), other parameters are also used to assess body weight, such as: waist-to-hip ratio, waist-to-height ratio, skinfold thickness, neck circumference, and waist circumference [8].

Advancements in technology and the discovery of new anthropometric parameters for quantifying excess adipose tissue, along with the need to better assess potential health risks associated with obesity, have led to the development of new measurement indices and analytical methods. These include body fat percentage, abdominal volume index, sagittal abdominal diameter, conicity index, visceral adiposity index, bioelectrical impedance analysis (BIA), and dual-energy X-ray absorptiometry (DEXA) [8].

The most widely used parameter is the Body Mass Index (BMI), which, according to the World Health Organization (WHO), defines overweight as a BMI between 25 and 29.9 kg/m^2^, and obesity as a BMI of 30 kg/m^2^ or higher [9].

As indicated by its unit of measurement, BMI is calculated as the ratio of a person’s weight to the square of their height. Although it is widely used and cost-effective, BMI shows a weak correlation with visceral fat (fat surrounding internal organs). Moreover, BMI does not distinguish between genders, and is inaccurate when applied to elderly individuals with cachexia or elite athletes with high muscle mass, due to its limitations in accurately reflecting body composition [10].

An update to the diagnostic criteria for obesity, which until 2014 relied exclusively on BMI, was introduced by the American Association of Clinical Endocrinologists (AACE). This update emphasized the need to include obesity-related complications as a parameter considered alongside the patient’s BMI. In other words, a patient with a BMI between 25 and 29.9 kg/m^2^, previously classified as merely overweight—regardless of whether any obesity-related complications were present—would now, under the new classification, be diagnosed with Class 1 obesity if a documented obesity-related complication is present. The rationale for incorporating complications into the classification system stems from the substantial annual healthcare costs associated with the management of obesity-induced diseases, as well as the significant burden of morbidity linked to this condition [11].

Other parameters—such as waist circumference—have also been analyzed and are considered more reliable indicators of visceral fat, making them important predictors of the multiple health complications associated with obesity. However, potential issues arise in individuals with a BMI over 40 kg/m^2^, due to inherent limitations in the measurement technique. Additionally, waist circumference cannot distinguish between subcutaneous fat and visceral fat. Unlike BMI, waist circumference varies by sex, with threshold values of 94 cm for men and 80 cm for women. Similarly to BMI, cut-off values differ for Asian and African populations compared to European populations. Although various recommendations exist, there is no universally accepted standard. Classically, waist circumference is measured midway between the lower margin of the last rib and the top of the iliac crest. Other methods involve measuring at the narrowest part of the waist, at the level of the navel, or along the line connecting the iliac crests [12]. The Lancet Commission on Diabetes and Endocrinology proposed an algorithmic model for the diagnosis of obesity. According to this model, a diagnosis of obesity requires the fulfillment of two criteria: the definition of obesity based on anthropometric measurements or body fat percentage, and the evidence of functional organ impairment secondary to obesity, or limitations in daily activities caused by excess body weight [13].

Obesity is associated with an increased risk of numerous conditions, including cardiovascular diseases (such as heart failure with reduced ejection fraction and atrial fibrillation), metabolic disorders (type 2 diabetes, dyslipidemia), genitourinary disorders (oligomenorrhea, polycystic ovary syndrome, urinary incontinence, hypogonadism), respiratory conditions (apnea, dyspnea), musculoskeletal disorders (joint pain, reduced range of motion), and even ocular pathologies [13].

Obesity is characterized by decreased testosterone, increased estrogen and a mild hypothyroid hormonal profile.

Androgens play a direct role in stimulating lipid secretion, maintaining glandular function, exerting anti-inflammatory effects through modulation of the local immune response, and preventing atrophy of the sebaceous glands. Consequently, in elderly individuals, as well as in patients with obesity, low androgen levels lead to the loss of trophic support for the Meibomian glands, contributing to the development of DES.

Estrogen exerts an inhibitory effect on lipid metabolism. The synthesis of lipid components within the Meibomian glands is diminished during menopause, a state characterized by low estrogen levels. Generally, low estrogen levels are considered a risk factor for the development of DES. However, some reports have suggested that elevated estrogen levels, such as those observed in obesity, may also predict altered Meibomian gland secretion.

A subclinical hypothyroid profile manifests through a reduced basal metabolic rate and weight gain. Therefore, patients present with the typical hormonal changes seen in obesity. Interestingly, obese patients with concurrent hyperthyroidism and an accelerated metabolic state may also develop DES. Patients with thyroid eye disease exhibit significant structural damage to the Meibomian glands, likely due to lymphocytic infiltration of the lacrimal gland, which leads to decreased tear production. This is compounded by the conformational changes associated with Graves’ orbitopathy, particularly marked exophthalmos, which increases the exposed ocular surface area. These combined mechanisms contribute to the onset of DES in this patient population [14].

## 2. Materials and Methods

### 2.1. Search Strategy

A search was conducted in the PubMed and Web of Science databases for articles published up to 1 October 2024, that included keywords from the two main groups.

The main keywords used to search for related articles were compiled in a separate Word document. The keywords were divided into two main groups: terms defining dry eye syndrome (Dry eye OR Ocular surface OR lacrimal film OR Tear Film OR TFOS OR DWES) and terms related to obesity or literature reports (Obesity OR High body mass OR High Body Index OR Report).

Within each category, the terms were combined using the Boolean operator OR to generate two main searches. The results of these searches were then combined using the Boolean operator AND to obtain the final search string: ALL = ((Obesity OR High body mass OR High Body Index OR Report) AND (Dry eye OR Ocular surface OR lacrimal film OR Tear Film OR TFOS OR DWES)).

Additional relevant articles were identified from the reference lists of the initially retrieved papers, based on the significance of their findings. It is important to note the difficulty in selecting articles that specifically address the chosen review topic, as relatively few studies have simultaneously evaluated obesity, tear film, and inflammation. For this reason, the initial search strategy focused separately on tear film alterations and obesity. Subsequently, articles examining systemic inflammation in the context of obesity and local inflammation in the context of DES were included. The final keyword search update was conducted on 25 January 2025. This narrative review was written between 1 October 2024 and 25 January 2025.

### 2.2. Inclusion and Exclusion Criteria

The reviewers applied the following inclusion criteria: (a) studies relevant to the chosen topic; (b) no restrictions were imposed regarding the publication date or language of the studies. The exclusion criteria included: (a) articles whose titles or abstracts were not deemed relevant to the scope of this review. There were no disagreements among the reviewers concerning the titles or content of the articles selected for inclusion.

### 2.3. Trial Flow/Selection Process

The search in the Web of Science database, using keywords combined with the Boolean operators OR and AND, yielded 163 results for the query: ALL = ((Obesity OR High body mass OR High Body Index OR Report) AND (Dry eye OR lacrimal film OR Tear Film OR TFOS OR DWES)). The results were refined using filters provided by the Web of Science search engine, specifically: Web of Science Category: Ophthalmology, Document Types: Review Article, Open Access, and Exact Search. Using the same search query in the PubMed database generated 48 results, after applying the following filters: Free full text, Humans, Review, Systematic Review, and searching within the title/abstract fields using the aforementioned keywords.

## 3. Results

Following the review of 211 articles based on their titles, abstracts, or duplication, 175 were excluded, and 36 were selected for full-text reading. Additionally, during the inclusion process, several other important articles were identified through separate searches. These were considered significant due to the relevance of the data they presented, even though they did not meet the initial inclusion criteria. As a result, 15 additional articles not retrieved in the original search were included in this narrative review. These were selected from the Google Scholar, Scopus, Medline, PubMed, and Web of Science databases.

The final number of articles evaluated in this review is 47.

**Figure 1:**
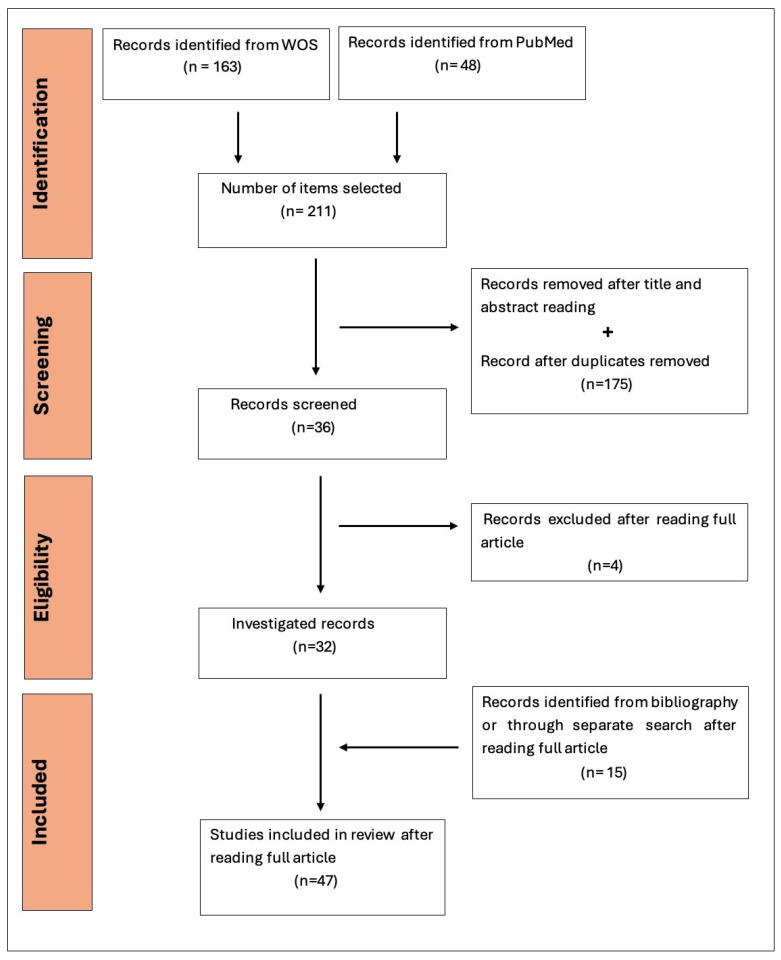
Flowchart of the literature search and study selection process.

Figure 1 summarizes the flow diagram of the search process. The overall quality of the included studies was assessed as good.

**Figure 2:**
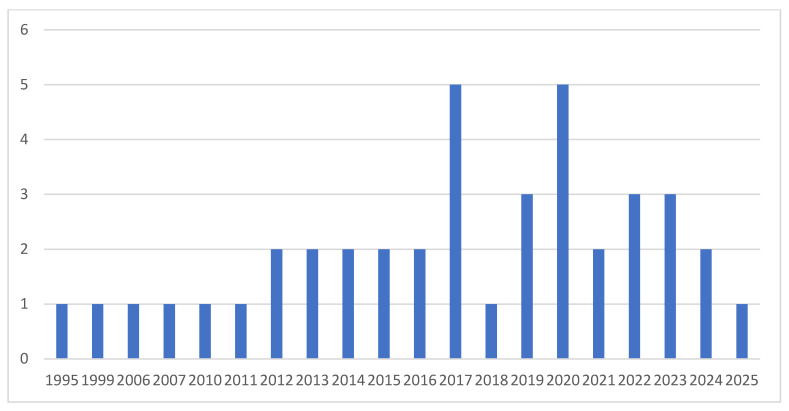
Distribution of articles included in the study by year of publication.

Figure 2 shows a gradual increase in the number of publications investigating the association between DES and obesity over the past 30 years.

### 3.1. Obesity and Ocular Pathology

The correlations between systemic disorders and obesity have been extensively studied and supported by numerous landmark studies. However, there is a relatively small number of studies that have specifically examined the development of obesity-induced ocular pathology. Certain statistical correlations have been recognized between obesity and the development of cataracts, intraocular hypertension, age-related macular degeneration, tear film dysfunction, and diabetic retinopathy. A few literature reviews have attempted to compile all available information on the relationship between obesity and ocular disorders up to the date of their publication [7].

### 3.2. Pathophysiological Links

It has been hypothesized that early-onset cataractogenesis in obese individuals may be triggered by elevated levels of oxidative stress, which is quantified systemically through increased levels of C-reactive protein (CRP). Among obese patients, the most commonly observed type of cataract is posterior subcapsular cataract [15].

Damage to the optic disc caused by persistently elevated intraocular pressure is known as glaucomatous optic neuropathy. Although some studies have identified a positive association between glaucoma and obesity, the exact pathophysiological mechanisms remain difficult to explain. Open-angle glaucoma is the most frequently reported type of glaucoma among individuals with weight disorders [16].

Most data in the literature suggest that oxidative stress within the trabecular meshwork disrupts aqueous humor outflow and leads to increased intraocular pressure. This occurs against the backdrop of a hyperandrogenic state typically associated with obesity [17]. Emerging theories also propose that individuals with excess adipose tissue experience intestinal dysbiosis, which alters gut permeability and leads to overactivation of Toll-Like Receptor 4 (TLR4) and determines neuronal inflammation and axonal neurodegeneration [18].

Persistent, dull headache and fluctuating visual deficits should alert the clinician to the possibility of pseudotumor cerebri. The currently accepted term for this pathological condition, first described by Nonne in 1914, is idiopathic intracranial hypertension (IIH). Papilledema results from increased pressure in the cerebrospinal fluid (CSF), often due to functional obstruction at the level of the venous sinuses. The association between a high body mass index (BMI) and ocular pathology is well-documented. As a result, standard recommendations include regular monitoring of patients with morbid obesity through automated perimetry and fundus examination, in order to prevent secondary blindness. Acute pharmacological treatment typically involves acetazolamide and hyperosmolar agents, alongside weight loss and, in some cases, bariatric surgery, which are the main strategies used to manage this condition [19,20].

Age-related macular degeneration (AMD) is a common condition in developed countries. Over time, retinal pigment epithelial (RPE) cells become less effective at facilitating the exchange of nutrients and oxidative waste between the retina and the bloodstream. This dysfunction leads to the accumulation of free radicals, lipids, the formation of drusen, cell death and migration, and ultimately the development of choroidal neovascularization. Smoking and excessive alcohol consumption are major risk factors for the onset and progression of AMD. Among obese patients, the waist-to-hip ratio is a better predictor than BMI for the risk of developing AMD. Since aging leads to sedentary behavior and weight gain, the positive association between obesity and AMD observed in studies becomes more understandable [7,21].

The association between diabetic retinopathy and obesity is well established. Retinal neovascularization is driven by vascular endothelial growth factor (VEGF), which is excessively produced in individuals with diabetes. While angiogenesis plays a critical role in the development of adipose tissue, obesity alone does not have the intrinsic capacity to stimulate VEGF-A production to the same extent [22].

Leptin is a peptide hormone secreted by adipocytes, playing a key role in signaling satiety. Through its actions at the hypothalamic level, leptin inhibits appetite. In obese individuals, a state of relative leptin resistance develops, requiring higher circulating levels of leptin to achieve neuroendocrine stimulation. Leptin is also essential for central insulin signaling, meaning that individuals with excess adipose tissue commonly exhibit acquired insulin resistance. Additionally, leptin has pro-inflammatory properties and is directly involved in the development of both hypertensive retinopathy and diabetic retinopathy in individuals with obesity [23].

Although diabetic retinopathy is commonly associated with increased BMI, several studies have paradoxically shown that underweight patients—those with a BMI below 20 kg/m^2^—have a threefold higher risk of developing retinal complications in the context of diabetes. This low BMI is most likely due to severe metabolic dysfunction induced by diabetes. However, a high BMI should not be considered protective, and weight reduction remains a strongly recommended measure [24].

### 3.3. Combined Therapy for Dry Eye Syndrome and Obesity

The tear film is influenced by dietary changes as well as systemic hydration status. Diet clearly plays a direct role in determining each individual’s caloric surplus or deficit. Over time, increasing evidence has supported the idea that the body’s hydration level regulates both systemic and local osmolarity, including that of the tear film [25]. Exposure to a dry environment increases the risk of developing DES. However, engaging in physical exercise even in such environments appears to have a protective effect on the tear film. These findings underscore the importance of regular physical activity as part of the therapeutic approach to managing DES [26].

Essential fatty acids are vital for human health but cannot be synthesized by the body, making dietary intake necessary. Omega-3 fatty acids are well-known, commonly promoted in weight-loss diets, and widely regarded as part of a healthy lifestyle. They are found in fatty fish and seeds, while omega-6 fatty acids are prevalent in vegetable oils. The Western diet typically leads to an excessive intake of omega-6, often up to 15 times higher than omega-3 intake, a ratio that has been linked to increased systemic inflammation. Clinical guidelines recommend reducing this ratio by at least a factor of three. Fish oil supplementation has been integrated into the therapeutic approach for DES. Omega-3 fatty acids promote the synthesis of Thromboxane A1 and Prostaglandin E1, which exert anti-inflammatory effects, and they inhibit pro-inflammatory cytokines such as IL-1, IL-2, and TNF-α. In contrast, omega-6 fatty acids stimulate the production of Thromboxane A2 and Prostaglandin E2, compounds considered to be pro-inflammatory. Omega-3 supplementation has been shown to alleviate both subjective symptoms and clinical signs of DES, and to improve outcomes in diagnostic assessments. However, excessive dosages may carry a risk of bleeding, particularly in patients with atrial fibrillation, hepatic insufficiency, or coagulation disorders [27,28].

It is therefore understandable why caloric restriction and weight loss can help alleviate certain symptoms of DES. Likewise, alcohol consumption should be kept moderate and aligned with international guidelines to prevent temporary DES episodes, even in otherwise healthy individuals [29]. As expected, cigarette smoke leads to lipid peroxidation within the tear film and disrupts ocular surface physiology due to the significant amount of free radicals introduced with each exhalation. In such cases, smoking cessation remains the primary therapeutic recommendation [30].

### 3.4. Management Strategies

Weight loss as a means of controlling systemic inflammation and improving or even preventing systemic complications associated with obesity is a key goal for any obese patient. Beyond the dietary adjustments needed to reduce caloric intake, which remain central to managing excess adipose tissue, any therapeutic plan for obesity should also include regular physical activity to help burn excess energy. A 2020 study by Hao Li, titled “Aerobic Exercise Increases Tear Secretion and Decreases Inflammatory Cytokines in Healthy Subjects,” examined the effects of indoor aerobic exercise on the tear film in healthy individuals. The study found that 30 min of treadmill exercise led to an increase in the volume of the lower tear meniscus, with a peak observed 10 min post-exercise and reduced inflammatory cytokines in the tear film (Interferon gamma (IFN-γ), TNF-α, IL-6, IL-10), with their lowest concentrations at 20 min post-exercise. It also showed a reduction in inflammatory cytokines in the tear film—namely interferon gamma (IFN-γ), TNF-α, IL-6, and IL-10—with the lowest concentrations observed 20 min after exercise. These values returned to baseline 60 min after exercise. The changes are likely due to the dilution of pro-inflammatory factors resulting from increased tear volume. Further studies are necessary to determine the influence of environmental factors on the effects of physical activity, as this study was conducted in a controlled indoor environment (temperature, humidity, wind). It would also be valuable to compare the lacrimal effects of physical activity in subjects with normal BMI versus those with elevated BMI [31].

In the 2019 study “Assessment of Tear Film in Subjects with a High Body Mass Index,” Alanazi SA et al. concluded that individuals with excess adipose tissue, as measured by BMI, exhibit alterations in tear film quality, although no significant differences were observed in the quantity of tear production [32].

Lifestyle modification was evaluated by Ismail AMA in the study “Effect of Aerobic Exercise Alone or Combined with Mediterranean Diet on Dry Eye in Obese Hypertensive Elderly”. The authors found a positive association between weight loss and the improvement of DES evaluation parameters. Apparently, caloric restriction contributes to the maintenance of normal lacrimal function in elderly patients and may even reverse certain pathophysiological mechanisms involved in the development of DES. There are still relatively few studies in the scientific literature that document the evolution of DES in relation to weight control [33].

An interesting association is that between DES, hypertension, and diabetes—all of which are well-known secondary pathologies induced by obesity. In the study “Prevalence and Associated Risk Factors of Dry Eye Disease in 16 Northern West Bank Towns in Palestine: A Cross-Sectional Study”, Shanti Y reported that within the study population, 20.9% of patients had both DES and hypertension, and 17% had both diabetes mellitus and DES. Although this study focused on the prevalence of DES in the northern West Bank population and did not assess patient weight or its relationship with DES, its findings regarding the co-occurrence of hypertension and diabetes with DES align with the positive associations between hypertension, obesity, and DES reported by Ismail AMA [34].

Although the study “Magnitude of Diabetes and Hypertension among Patients with Dry Eye Syndrome at a Tertiary Hospital of Riyadh, Saudi Arabia—A Case Series” by Al Houssien AO was conducted on a small sample of 62 patients and lacked a control group, it identified a positive association between obesity, DES, and other potential metabolic complications, supporting the findings of Ismail AMA. Specifically, Al Houssien AO reported that among the 62 patients, 37% had DES, 48.5% had hypertension, 47.1% suffered from diabetes, and 55.9% had dyslipidemia. Despite environmental and climatic factors specific to the Middle East—such as low humidity, extreme heat, and prolonged air conditioning use, which are known to contribute to higher incidence and prevalence of DES—it is evident that metabolic syndrome plays a key role in triggering this ocular surface disease [35].

The connection between DES and sedentary behavior was examined by Kawashima M in the study “The Association between Dry Eye Disease and Physical Activity as well as Sedentary Behavior: Results from the Osaka Study.” This research investigated the positive correlation between DES and physical activity levels in employees who work extensively with video display terminals (VDTs). Sedentarism is linked to weight gain due to reduced energy expenditure. Although the study did not analyze participants’ weight, it focused on quantifying physical activity using weekly metabolic equivalent units (METs). It demonstrated that the mere lack of physical activity, combined with prolonged screen time, significantly contributes to DES—ultimately reducing workplace productivity. Notably, 12% of employees who spend more than 8 h daily in front of VDTs were found to have DES [36].

There is a well-documented positive association between depression, anxiety, and DES. The specific symptoms of DES—such as eye heaviness, grittiness, dryness, itching, and redness—significantly affect quality of life. Patients often require long-term treatment involving artificial tears, which can disrupt daily activities. Since artificial tear drops are not reimbursed, the cost of medication may further burden individuals already facing financial difficulties. In the study “Associations between Subjective Happiness and Dry Eye Disease: A New Perspective from the Osaka Study”, Kawashima M examined the relationship between DES and subjective happiness, using the Lyubomirsky and Lepper happiness scale. The findings revealed that individuals facing economic, workplace, or family-related stress scored lower on the happiness scale. Moreover, they reported subjective symptoms that were disproportionately intense relative to their objective clinical signs of DES [37].

Unlike the adult population, the study “Obesity Is Associated with Oxidative Stress Markers and Antioxidant Enzyme Activity in Mexican Children” reported increased enzymatic activity of catalase and glutathione peroxidase in obese children compared to their normal-weight peers. This paradox may be related to age-associated hormonal changes or a hormonal protective response to oxidative stress. Additionally, the study “Obesity, Physical Fitness, and Inflammatory Markers in Polish Children” suggests that female pediatric patients show greater resistance to metabolic complications associated with obesity, possibly due to sex-specific physiological factors [38].

For further details regarding the studies discussed in this section, please refer to Table 2.

## 4. Discussion

### 4.1. Systemic and Local Oxidative Stress Analysis

C-reactive protein (CRP) is widely recognized as a nonspecific marker of systemic inflammation. The study “C-Reactive Protein Levels and Tear Function Parameters” explored CRP levels within the tear film to compare systemic and local sites of inflammatory activity. However, the findings did not demonstrate a significant positive correlation between systemic and ocular surface inflammation [39].

Lifestyle changes, particularly dietary patterns, significantly influence systemic inflammation. Since its initial investigation in the 1960s, the Mediterranean diet—rich in unsaturated fats—has been reported as ideal for weight loss and maintaining low levels of systemic inflammation. In contrast, the Dietary Approaches to Stop Hypertension (DASH) diet, commonly used in the United States, is low in fat but rich in minerals, with the exception of sodium. On the other hand, Western diets, which are typical in today’s modern, fast-paced society, are characterized by high intakes of saturated fats, alcohol, and carbohydrates. These contribute to a state of acute postprandial inflammation and induce alterations in the gut microbiome that sustain chronic inflammation over time [40].

To assess the impact of nutrition on inflammatory status, the Dietary Inflammatory Index (DII) was developed in 2014. This index is based on the body’s inflammatory response, measured by markers such as C-reactive protein (CRP), tumor necrosis factor-alpha (TNF-α), and interleukins (IL-1β, IL-4, IL-6, IL-10), in reaction to the intake of various dietary components. The DII assesses 45 dietary parameters, resulting in a score that ranges from maximum pro-inflammatory (+7.98) to maximum anti-inflammatory (−8.87), depending on the nutritional profile evaluated. This is documented in the study “Dietary Inflammatory Index and Anthropometric Measures of Obesity in a Population Sample at High Cardiovascular Risk from the PREDIMED (PREvención con DIeta MEDiterránea) Trial” [41].

The balance of the gut microbiome is also influenced by dietary patterns. The dominant microbial phyla are Bacteroidetes (Gram-negative bacteria) and Firmicutes (Gram-positive bacteria), with Bacteroidetes typically being more prevalent. However, a shift toward a higher Firmicutes-to-Bacteroidetes ratio, often caused by poor dietary habits, can disrupt gut homeostasis. This imbalance leads to hyperactivation of Toll-Like Receptor (TLR) pathways and increased intestinal permeability. The translocation of lipopolysaccharides (LPS) into the bloodstream triggers a state of metabolic endotoxemia, which subsequently activates transcription factors such as Nuclear Factor-kappa B (NF-κB). This transcription factor promotes the expression of pro-inflammatory genes, resulting in elevated secretion of inflammatory cytokines including IL-1, IL-6, TNF-α, and IFN-γ [42].

The gut microbiome plays a central role in regulating systemic inflammation. Commensal bacteria exert immunomodulatory effects through the production of short-chain fatty acids, such as butyric acid.

By maintaining the integrity of the intestinal barrier, the gut microbiota inhibits the translocation of activated T and B lymphocytes, as well as pro-inflammatory cytokines, via the lymphatic vessels to the ocular surface. Furthermore, studies have documented alterations in the gut microbiome of patients with dry eye disease associated with Sjögren’s syndrome. These patients exhibit a reduced concentration of commensal bacteria that produce butyric acid, as well as of bacteria with inhibitory effects on regulatory T cells, which are involved in suppressing inflammation driven by Th17 lymphocytes.

These findings suggest the existence of a pathophysiological regulatory axis between two distinct systems, the ocular and gastrointestinal systems, both implicated in the control of local and systemic inflammation [43].

In obesity, adipose tissue is a key driver of systemic inflammation. As more fat accumulates, either due to low energy expenditure or excess caloric intake, the resident macrophage population within adipose tissue shifts. Physiologically, adipose tissue contains predominantly M2 macrophages, which are anti-inflammatory. However, in obesity, these are gradually replaced by bone marrow-derived M1 macrophages, which promote the secretion of pro-inflammatory cytokines [40].

Obesity is also a core component of the metabolic syndrome, a condition defined by chronic low-grade systemic inflammation. The link between systemic inflammation and obesity is the excess adipose tissue itself. While adiposity was once viewed simply as inert storage, it is now recognized as an active endocrine organ that secretes hormones regulating both satiety and inflammatory responses. One such hormone is adiponectin, secreted by adipocytes. It has antioxidant properties and paracrine anti-inflammatory effects by recruiting M2 macrophages and metabolic effects, such as reducing lipid synthesis, and inhibiting gluconeogenesis (glucose production from non-carbohydrate sources). These roles are outlined in the study “Obesity and Inflammation: The Linking Mechanism and the Complications”. However, as previously mentioned, adiponectin alone cannot maintain the balance of systemic inflammation in obesity. Other adipokines, such as leptin, IL-6, TNF-α, and monocyte chemoattractant protein-1 (MCP-1), are pro-inflammatory and contribute to the state of chronic inflammation observed in obese patients [44].

Another line of evidence regarding the role of oxidative stress in DED is provided by the article “Toll-like receptor 4 expression and oxidative stress in ocular rosacea”. Elevated levels of TLR4 are associated with the release of pro-inflammatory factors. In this context, Yesilirmak N. and colleagues analyzed oxidative stress markers in the serum and tears of 60 patients, including 40 with ocular rosacea. Patients with ocular rosacea demonstrated increased TLR4 expression both at the ocular surface and in peripheral blood [45].

Additionally, the study “Evaluation of Ocular and Systemic Oxidative Stress Markers in Ocular Rosacea Patients” conducted by Yesilirmak N. et al. on 120 patients, of whom 90 had ocular rosacea, suggested the presence of reduced antioxidant levels in these individuals. It is hypothesized that such conditions may create a permissive environment for the development of ocular rosacea [46].

Table 3 highlights a selection of studies that have suggested the presence of systemic inflammation associated with obesity.

### 4.2. Obesity-Related DES

One of the most significant pandemics of the twenty-first century is metabolic disease. Obesity is now known to be a systemic risk factor for diseases of the heart, endocrine system, lungs, kidneys, oncology, and gastrointestinal tract. DES has historically been divided into two primary groups: evaporative DES and aqueous-deficient DES. As shown in Table 1, the classification of DES has changed over time, starting with the original framework put forth by Lemp in 1995 and ending with the updated model by Tsubota et al. in 2020. The current classification of dry eye syndrome needs to be reexamined in light of new data [1,5].

Consequently, we suggest adding a new subtype of DES called obesity-related DES, which has a unique clinical profile and multifactorial pathophysiology.

By changing the quality of lipids secreted by the meibomian glands and causing a chronic low-grade systemic inflammatory state, obesity may be a contributing factor to both subtypes of DES. According to Ellulu et al. proinflammatory cytokines like TNF-α and IL-6 are elevated in obese patients, which leads to systemic inflammation. According to Grosso et al. dietary interventions such as the adoption of DASH or Mediterranean diets may be able to modulate this process.

Obesity-related DES is a term that should be used to describe the complex pathophysiological mechanisms through which obesity impacts the amount and quality of tears [40,44].

At the intersection between ocular surface disease and chronic systemic pathology, this newly proposed DES subtype represents an emerging research domain in which the eye becomes a primary target organ of systemic imbalance.

Early detection of these ocular symptoms enables patients with obesity-related DES to undergo lifestyle changes in addition to receiving symptomatic treatment with artificial tear supplements. Alanazi et al. showed a link between elevated BMI and tear film instability, as shown in Table 2 [32].

Furthermore, participants’ signs and symptoms of DES significantly improved after completing the six-month intervention suggested by Ismail et al. which involved a Mediterranean diet and regular exercise three times a week [33].

This concept opens a new transdisciplinary research direction at the crossroads of ophthalmology, endocrinology, and nutrition, where the eye emerges not only as an organ affected by obesity but also as a potential biomarker of systemic inflammation and metabolic dysfunction.

### 4.3. Strenghts and Limitations

The key strength of this study lies in its attempt to correlate systemic inflammation with tear film inflammation in patients with obesity, through the detailed analysis of 41 studies that captured the key characteristics of both pathological processes. However, there are several limitations associated with this review.

First, a significant number of articles—9 out of 41—were independently searched and included outside of the initial Boolean keyword strategy. The use of Boolean operators did not yield a sufficiently comprehensive or relevant data flow. Various keyword combinations were tested, but only the approach presented in this review proved viable.

Second, there is a notable lack of studies directly investigating the relationship between tear film inflammation and systemic inflammation. Most of the existing literature does not establish direct correlations between the two. Therefore, future research is necessary to quantitatively analyze both systemic and ocular surface inflammation, particularly in the context of obesity.

## 5. Conclusions

Obesity is a complex, often recurrent metabolic disease that affects virtually all systems of the body. While the associations between obesity and alterations in tear quality or quantity are well-recognized, most investigations have approached this strictly from an ophthalmological perspective.

DES tends to emerge as a positive finding in relation to obesity, yet studies examining the role of systemic inflammation induced by obesity and its potential to trigger oxidative stress at the ocular surface remain scarce. DES is a multifactorial condition defined by a loss of tear film homeostasis. Though typically viewed as a localized ocular disorder, it can significantly impair quality of life.

Undoubtedly, the coming years will witness a surge in research focused on the tear film. We believe that the analysis of tear biomarkers holds promise not only for refining the classification of major systemic diseases, but also for potentially enabling their early screening through a simple tear analysis.

Addressing systemic inflammation induced by obesity requires further research and a multidisciplinary management approach to ensure effective prevention of obesity-related complications and to improve overall patient outcomes.

## Figures and Tables

**Table 1:** Advances in dry eye syndrome diagnosis.

Author and Year	Report	Key Findings
Lemp (1995) [1]	NEI/Industry Workshop	The first definition of DES classified the condition into aqueous tear deficiency and evaporative state. This condition is considered a disorder of the tear film.
Lemp et al. (2007) [2]	Tear Film & Ocular Surface Society Dry Eye Workshop I (TFOS DWES I)	The revised definition of DES introduces a classification into aqueous-deficient and evaporative types, reflecting a paradigm shift that also acknowledges dry eye as a disease rather than merely a disorder.
Bron et al. (2017) [4]	Tear Film & Ocular Surface Society Dry Eye Workshop II—Pathophysiology (TFOS DWES II)	Update to the definition introduced by DEWS I DEWS II emphasizes the spectrum of DES presentations, encompassing aqueous-deficient, mixed, and evaporative types. The revised algorithm also accounts for asymptomatic cases with clinical relevance as well as symptomatic cases lacking observable clinical signs.
Tsubota et al. (2020) [5]	Asia Dry Eye Society (ADES)	ADES introduces a new element by adding, alongside evaporative and aqueous-deficient causes, a third category: Dry eye resulting from decreased wettability of the ocular surface.

NEI/Industry Workshop—National Eye Institute/Industry Workshop, TFOS DWES I—Tear Film & Ocular Surface Society Dry Eye Workshop I, TFOS DWES II—Tear Film & Ocular Surface Society Dry Eye Workshop II, ADES—Asia Dry Eye Society.

**Table 2:** Studies investigating the impact of lifestyle on DES.

Author and Year	Participants and Method	Key Findings
Walsh et al. (2012) [25]	Observational cross-sectional study involving 111 patients.	Suboptimal hydration contributes to tear film hyperosmolarity and increases the risk of developing DES.
Alanazi (2019) [32]	Prospective study involving 20 male patients with elevated BMI. Both the quantity and quality of tears were assessed.	Patients with elevated BMI exhibit qualitative alterations in tear composition, while tear quantity remains within normal limits.
Peart et al. (2020) [26]	Prospective study involving 12 healthy individuals. The tear response was evaluated following 20 min of physical exercise under low humidity conditions (20%).	Physical exercise has a beneficial effect on the tear film, even in environmental conditions that predispose to DES.
Li et al. (2020) [31]	Prospective cross-sectional study involving 43 patients. The impact of aerobic exercise—defined as running at 6 km/h for 30 min on a treadmill—was analyzed under controlled conditions of temperature (22 °C) and humidity (60%).	Indoor aerobic exercise can increase tear secretion and reduce inflammation in the tear film in healthy individuals.
Magno et al. (2021) [29]	Group study involving 77,145 participants between 2014 and 2018. The Women’s Health Study (WHS) questionnaire was used to assess DES and the Food Frequency Questionnaire (FFQ) was used to evaluate alcohol consumption.	Alcohol consumption increases the risk of developing DES in women, while paradoxically showing a protective effect in men at moderate intake levels (10 g/day).
Tariq et al. (2022) [30]	Meta-analysis including 160,217 patients across 22 studies published between 2000 and 2021.	Smoking shows a paradoxical protective effect against DES. However, individuals who quit smoking tend to develop DES. Despite this, the systemic health risks associated with smoking far outweigh its potential protective role in DES.
Ismail et al. (2023) [33]	Randomized controlled clinical trial involving 60 patients, assessing the effect of high-intensity aerobic exercise with and without a Mediterranean diet on DES.	A Mediterranean diet combined with high-intensity aerobic exercise (30 min, three times per week for six months) has been shown to improve both clinical signs and subjective symptoms in patients with DES.

BMI—Body Mass Index, WHS—Women’s Health Study, FFQ—Food Frequency Questionnaire.

**Table 3:** Studies Evaluating Systemic and Local Oxidative Stress in Obesity.

Author and Year	Participants and Method	Key Findings
Crane et al. (2013) [39]	Prospective study that evaluated 263 patients.	C-reactive protein (CRP) is not a reliable indicator for evaluating inflammation in the tear film.
Ruiz-Canela et al. (2015) [41]	Multicenter clinical trial conducted on a group of 7447 patients.	The Dietary Inflammatory Index (DII) serves as a tool for quantifying systemic inflammation induced by dietary intake.
Ellulu et al. (2017) [44]	Literature review article.	High levels of interleukin-6 (IL-6) and tumor necrosis factor-alpha (TNF-α) are strongly correlated with systemic inflammation associated with obesity.
Grosso et al. (2022) [40]	Literature review article.	Both the Mediterranean diet and the DASH diet are linked to a reduction in systemic inflammatory markers.
Pezzino et al. (2023) [42]	Literature review article.	A gut microbiome marked by an increased abundance of Firmicutes and a reduced presence of Bacteroides contributes to the promotion of systemic inflammation.

CRP—C Reactive Protein, DASH—Dietary Approaches to Stop Hypertension, TNF-α—Tumor Necrosis Factor α, IL-6—Interleukin 6.

## Data Availability

The original contributions presented in this study are included in the article. Further inquiries can be directed to the corresponding authors.

## Abbreviations

The following abbreviations are used in this manuscript:

ADES	Asia Dry Eye Society
DASH	Dietary Approaches to Stop Hypertension
DEXA	Dual-Energy X-ray Absorptiometry
DII	Dietary Inflammatory Index
AMD	Age-Related Macular Degeneration
FFQ	Food Frequency Questionnaire
IL	Interleukin
BMI	Body Mass Index
MET	Metabolic Equivalent
NEI/Industry Workshop	National Eye Institute/Industry Workshop
NF-kB	Nuclear Factor-kappa B
CRP	C-Reactive Protein
DES	Dry Eye Syndrome
TFOS DWES II	Tear Film & Ocular Surface Society Dry Eye Workshop II
TLR4	Toll-Like Receptor 4
VEGF	Vascular Endothelial Growth Factor
WHO	World Health Organisation
WHS	Women’s Health Study
